# Dihydroavenanthramide D Enhances Skin Barrier Function through Upregulation of Epidermal Tight Junction Expression

**DOI:** 10.3390/cimb46090547

**Published:** 2024-08-23

**Authors:** Jiye Park, Jae Young Shin, Daehyun Kim, Seung-Hyun Jun, Eui Taek Jeong, Nae-Gyu Kang

**Affiliations:** LG Household & Health Care (LG H&H) R&D Center, 70 Magokjoongang 10-ro, Gangseo-gu, Seoul 07795, Republic of Korea; jyuy0228@lghnh.com (J.P.); sjy2811@lghnh.com (J.Y.S.); daehyun@lghnh.com (D.K.); junsh@lghnh.com (S.-H.J.); etjeong@lghnh.com (E.T.J.)

**Keywords:** skin barrier, skin sensitivity, avenanthramides, dihydroavenanthramide D, anti-aging

## Abstract

Skin barrier dysfunction and thin epidermis are hallmarks of sensitive skin and contribute to premature aging. Avenanthramides are the primary bioactive components of colloidal oatmeal, a commonly used treatment to enhance skin barrier function. This study investigated the relationship between skin barrier function and epidermal characteristics and explored the potential of dihydroavenanthramide D (dhAvD), a synthetic avenanthramide, to improve the skin barrier. We observed a significant correlation between impaired skin barrier function and decreased epidermal thickness, suggesting that a weakened barrier contributes to increased sensitivity. Our in vitro results in HaCaT cells demonstrated that dhAvD enhances keratinocyte proliferation, migration, and tight junction protein expression, thereby strengthening the skin barrier. To mimic skin barrier dysfunction, we treated keratinocytes and full-thickness skin equivalents with IL-4 and IL-13, cytokines that are implicated in atopic dermatitis, and confirmed the downregulation of tight junction and differentiation markers. Furthermore, dhAvD treatment restored the barrier function and normalized the expression of key epidermal components, such as tight junction proteins and natural moisturizing factors, in keratinocytes treated with inflammatory cytokines. In the reconstructed human skin model, dhAvD promoted both epidermal and dermal restoration. These findings suggest that dhAvD has the potential to alleviate skin sensitivity and improve skin barrier function.

## 1. Introduction

The disruption of the skin barrier leads to physiological changes including increased skin sensitivity, dryness, itchiness, and premature aging [1,2,3]. Moreover, skin barrier disruption triggers and exacerbates the sensitization caused by the invasion of microbes, allergens, and irritants [4]. The infiltration of sensitizers into the epidermis triggers the activation of type 2 inflammatory cytokines, such as IL-4, IL-5, and IL-13, which in turn accelerate the allergic response [5]. The impairment of epidermal integrity induced by Th2-mediated sensitization contributes to the exacerbation of dryness and itchiness. In particular, several factors including leaky tight junctions, loosely packed lipids, and activated proteases contribute to diminished skin capacity to retain hydration [6]. The expression of filaggrin and natural moisturizing factors (NMFs) is decreased by Th2 cytokines [7,8,9], repetitive scratching, detergent use, low humidity, exogenous or endogenous protease, air pollution, and corticosteroids [10,11]. Th2 cells contribute to itching in two ways. First, Th2-mediated inflammation leads to the loosening of tight junctions in the epidermis, allowing sensory nerve endings to penetrate the upper layers of the skin and become sensitized to itch-inducing stimuli [12,13]. Second, Th2 cells produce cytokines such as IL-4 and IL-13, which enhance the responsiveness of sensory neurons to histamine, a key mediator of itch sensation [14,15].

Premature aging due to barrier dysfunction is closely associated with increased skin inflammation. Aging-associated changes in epidermal barrier function contribute to increased epidermal permeability, which cannot be easily normalized [16,17]. Sustained impairment of skin barrier function leads to the accumulation of cutaneous inflammatory cytokines that cause inflammation [18,19].

**Figure 1:**
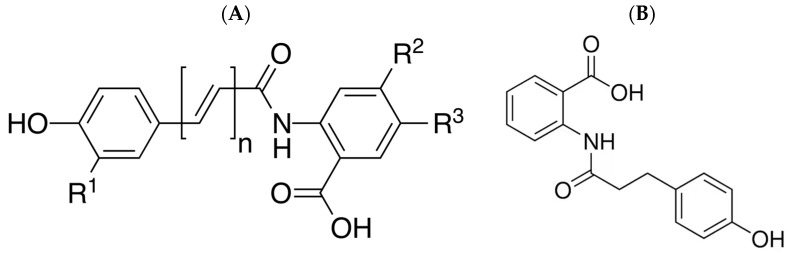
Comparison of the chemical structures of avenanthramide (**A**) and dihydroavenanthramide D (dhAvD, (**B**)).

Among the various approaches used to enhance skin barrier function, colloidal oatmeal has emerged as a promising natural remedy with a long history of traditional use [20,21]. Colloidal oatmeal, which is FDA approved as an over-the-counter (OTC) ingredient for the treatment of eczema, possesses anti-inflammatory, antioxidant, and skin-calming properties [22]. Avenanthramides, a group of phenolic alkaloids and amide esters comprising several anthranilic and cinnamic acid structures, are the primary bioactive components of colloidal oatmeal (Figure 1). Avenanthramides exhibit potent anti-inflammatory and antioxidant effects and are known to strengthen the skin barrier function and alleviate skin irritation [23]. Dihydroavenanthramide D (dhAvD) is a synthetic avenanthramide, and recent studies have suggested its potential benefits as an antioxidant and anti-inflammatory molecule [24,25] (Figure 1). The most significant structural difference between avenanthramide and dihydroavenanthramide D lies in the presence or absence of a double bond within their molecular structure. Dihydroavenanthramide D lacks the double bond found in avenanthramide, as it has been saturated with two additional hydrogen atoms. This double bond renders avenanthramide more susceptible to oxidation and other chemical reactions, making dihydroavenanthramide D chemically more stable. While the presence or absence of this double bond may influence the biological activities of these compounds, the exact mechanisms underlying these differences are not yet fully understood.

In this study, we investigated the barrier-enhancing effects of dhAvD and aimed to elucidate its underlying mechanisms. We established an in vitro model of atopic dermatitis-like barrier disruption by treating human keratinocytes with IL-4 and IL-13, two key cytokines involved in Th2 inflammation. Furthermore, we conducted a clinical trial to evaluate the efficacy of dhAvD in alleviating skin sensitivity by enhancing barrier function in individuals with sensitive skin.

## 2. Materials and Methods

### 2.1. Materials

Synthetic avenanthramide and dihydro-avenanthramide D (PHL85735) were purchased from Merck (Darmstadt, Germany). Recombinant human interleukin 4 and interleukin 13 were purchased from R&D Systems (Minneapolis, MN, USA).

### 2.2. Cell Culture

Human epidermal keratinocyte cell lines (HaCaT cells) were cultured in Dulbecco’s modified Eagle medium (DMEM, Gibco, Waltham, MA, USA) supplemented with 10% fetal bovine serum (FBS, Gibco, Waltham, MA, USA), 100 U/mL penicillin and 100 µg/mL streptomycin (Gibco, Waltham, MA, USA). Cells were maintained in a humidified incubator at 37 °C with 5% CO_2_.

### 2.3. Cell Viability Assay

Cell viability was measured using the CCK-8 assay (Dojindo, Rockville, MD, USA) following the manufacturer’s protocol. Briefly, HaCaT cells (3 × 10^3^ cells/well) were seeded in 96-well plates and cultured for 24 h. HaCaT cells were treated with various concentrations of dhAvD and incubated for 24 h at 37 °C with 5% CO_2_. Next, 10 μL of CCK-8 reagent was added to each well, and the mixture was incubated at 37 °C for an additional 2 h. Subsequently, the absorbance of each well was determined at 450 nm using an Epoch micro plate spectrophotometer (BioTek, Winooski, VT, USA).

### 2.4. Scratch Assay

HaCaT cells (5 × 10^4^ cells/well) were seeded in a Culture-Insert 2 Well in a μ-plate 24 (ibidi, Munich, Germany) and incubated for 24 h. After removing the insert, dhAvD was added at appropriate concentrations. The gap was photographed at 0 and 16 h with an EVOS™ FL Auto2 Imaging System (Thermo Fisher Scientific, Waltham, MA, USA). The gap area was analyzed using the ImageJ/Fiji^®^ (Version: 1.51j8) plugin called Wound_healing_size_tool (Version: 1.0, NIH, Bethesda, MD, USA).

### 2.5. mRNA Analysis

HaCaT cells (3 × 10^3^ cells/well) were seeded in 6-well plates and cultured for 24 h. The cells were then treated with IL-4/IL13 and dihydroavenanthramide D at various concentrations for 24 h. Total RNA was isolated using an RNA isolation kit (Qiagen, RNeasy kit, Germantown, MD, USA), according to the manufacturer’s instructions. cDNA was synthesized from RNA by reverse transcription using a cDNA synthesis kit (Bioneer, AccuPower^®^ RT PreMix & Master Mix, Daejeon, Republic of Korea). Gene expression was quantified by real-time PCR (qRT-PCR) with Taqman^TM^ One-step RT-PCR master mix reagent (Life Technologies, TaqMan™ Gene Expression Master Mix, Carlsbad, CA, USA). TaqMan probes for real-time PCR were purchased from Thermo Fisher Scientific (Waltham, MA, USA). The PCR reactions were performed on a StepOnePlus™ Real-Time PCR System (Thermo Fisher Scientific, Waltham, MA, USA) following the manufacturer’s protocol. The resulting data were analyzed with the software included in the PCR machine (delta-delta Ct method).

### 2.6. Three-Dimensional Human Reconstructed Skin Equivalent Analysis

Reconstructed human full thickness (epidermis and dermis) skin equivalent was prepared in Neoderm^®^-ED 12-well plates from TEGO science (Seoul, Republic of Korea). The 3D skin equivalents were stimulated for 3 days in an air–liquid interface culture with 15 ng/mL IL-4 and 15 ng/mL IL-13 to induce an atopic dermatitis-like morphology. Next, 40 μg/mL of dhAvD was added and co-treated with IL-4/IL-13 media for 3 days to the apical side of the air–liquid interface, which was replaced every day to evaluate barrier enhancement effects. Each treated tissue sample was fixed in 4% formaldehyde and embedded in paraffin. After deparaffinization and rehydration [26], 3 μm thick tissue slides were prepared. For H&E staining, hematoxylin and eosin (Merck, Darmstadt, Germany) were treated sequentially to tissue for 2 h. For immunostaining, after the antigen retrieval and permeabilization steps [27], the sectioned tissues were blocked with PBS containing 5% FBS and 1% BSA. After consecutive incubation with primary antibodies (anti-MMP1; ab52631, anti-collagen; ab270993, anti-filaggrin; ab81468, anti-involucrin; ab53112, 1:200 dilution, Abcam, Cambridge, UK/anti-CLDN; PA516875, anti-ZO1; #33-9100, 1:200 dilution, Thermo Fisher Scientific, Waltham, MA, USA) at 4 °C for 12 h, Alexa Flour 488 or Alexa Flour 594-conjugated secondary antibodies (1:1000 dilution, Abcam, Cambridge, UK) were added at room temperature for 1 h, and nuclei were stained with DAPI (1:2000 dilution, Thermo Fisher Scientific, Waltham, MA, USA) in the dark for 10 min. High-resolution fluorescence images were obtained using an EVOS^TM^ FL Auto2 Imaging System (Thermo Fisher Scientific, Waltham, MA, USA).

### 2.7. Clinical Assessment Design

The study was conducted in accordance with the ethical standards of the Declaration of Helsinki and was approved by the Institutional Review Board of LG Household & Health Care Ltd. (approval number NOTICE-LGHH-20240530-AB-04-01). All participants were informed of the purpose of the study and provided written informed consent prior to participation. A total of 39 healthy subjects from South Korea aged 34 ± 5 years were enrolled in this study. Skin barrier parameters (transepidermal water loss (TEWL), epidermal thickness, and sensitivity) were also evaluated. Baseline skin characteristics were measured in all volunteers. Among the participants, six volunteers who showed severe sensitivity were tested for the dhAvD complex to elucidate skin barrier recovery function. Details of the experiments are supplied in Section 2.8 and Section 2.9.

### 2.8. Glycolic Acid Stinging Test

Glycolic acid (Corbion, Amsterdam, The Netherlands) was diluted in a vehicle composed of distilled water, a thickener, and a pH adjuster to obtain a working concentration of 10%. Following the application of 10% glycolic acid (pH 4.0) to the folds of the nasolabial triangle, the participants self-evaluated the intensity of the sting sensation on a 4-point scale (0 = no stimulation, 1 = mild stimulation, 2 = moderate stimulation, and 3 = severe stimulation).

### 2.9. Evaluation of TEWL and Epidermal Morphology

Before the measurements, the participants cleaned their faces and acclimated for 20 min in an air-conditioned room (temperature 22 ± 2 °C; relative humidity 50 ± 10%). Skin measurements were performed on the cheek area of the face. The TEWL was measured using a Tewameter^®^ TM 300 (C + K electronic GmbH, Vechta, Germany), which is an open-chamber device suitable for measuring the water flux density diffusing from the skin sites. The TEWL is an indispensable parameter for evaluating the water barrier function of the skin. Epidermal thickness and density were measured using a high-frequency skin ultrasound device (Dermascan C, Cortex Technology, Aalborg, Denmark). The epidermal thickness was measured as the distance from the outermost epidermal layer to the dermal-epidermal junction.

### 2.10. Data and Statistical Analysis

All experimental data are presented as the mean ± standard deviation (S.D.) of at least five independent experiments, unless otherwise indicated. Statistical significance was determined using Student’s *t*-test. Differences were considered statistically significant at *p* < 0.05.

## 3. Results

### 3.1. Epidermal Thickness and TEWL Were Correlated with Skin Sensitivity

**Figure 2:**
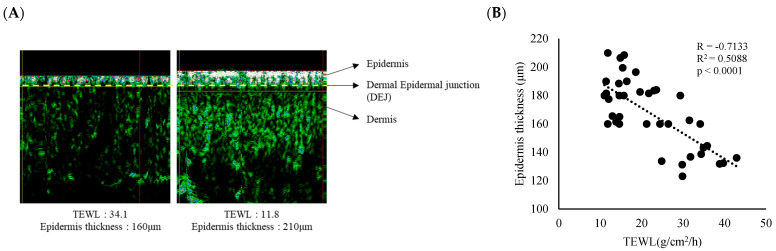
Correlation between impaired skin barrier function and epidermal characteristics. (**A**) Representative ultrasound images of thin and thick epidermis along with their corresponding TEWL values are shown. (**B**) TEWL and epidermal thickness are plotted. A significant correlation can be observed between TEWL and epidermal thickness (R = −0.7133, R^2^ = 0.5088, *p* < 0.0001). Data are expressed as the mean ± SEM. Correlation (R), R-squared (R^2^), and *p*-value were calculated using ANOVA regression analysis to assess the relationship between TEWL values and each variable of epidermis thickness.

To investigate the relationship between skin barrier function and epidermal characteristics, TEWL and epidermal thickness were measured in healthy participants. A strong inverse relationship was observed between TEWL and epidermal thickness (R = −0.7133, R^2^ = 0.5088, *p* < 0.0001), indicating that individuals with impaired skin barrier function tended to have thinner epidermal layers (Figure 2A,B). A questionnaire-based assessment was conducted to evaluate subject sensitivity to glycolic acid. The same subjects were administered 10% glycolic acid on their cheeks and categorized into three groups based on their self-reported sting score (0–4): none (0), mild (1), and severe (2–3). We observed that the TEWL of the cheek area in the group that experienced severe stinging with 10% glycolic acid was significantly higher (32.6 g/cm^2^/h) than that of the group that did not experience stinging (14.1 g/cm^2^/h). Additionally, the average epidermal thickness in the severe group was significantly thinner (147.3 μm) compared to that in the none group (185.8 μm) (Table 1). The overall demographic information is presented in Table 2. Based on these results, we confirmed that damaged skin barriers are generally characterized by a thinner epidermis, making them more susceptible to stinging stimuli.

### 3.2. Barrier-Enhancing Effects of Dihydroavenanthramide D on Cultured Human Keratinocytes

Based on these experiments, we hypothesized that epidermal thickening could be a promising strategy for improving skin barrier function and alleviating skin sensitivity. Since the stratum corneum and tight junctions are the main factors that strengthen physical barriers, we investigated the effects of dhAvD derived from colloidal oatmeal, focusing on these two main factors [26].

**Figure 3:**
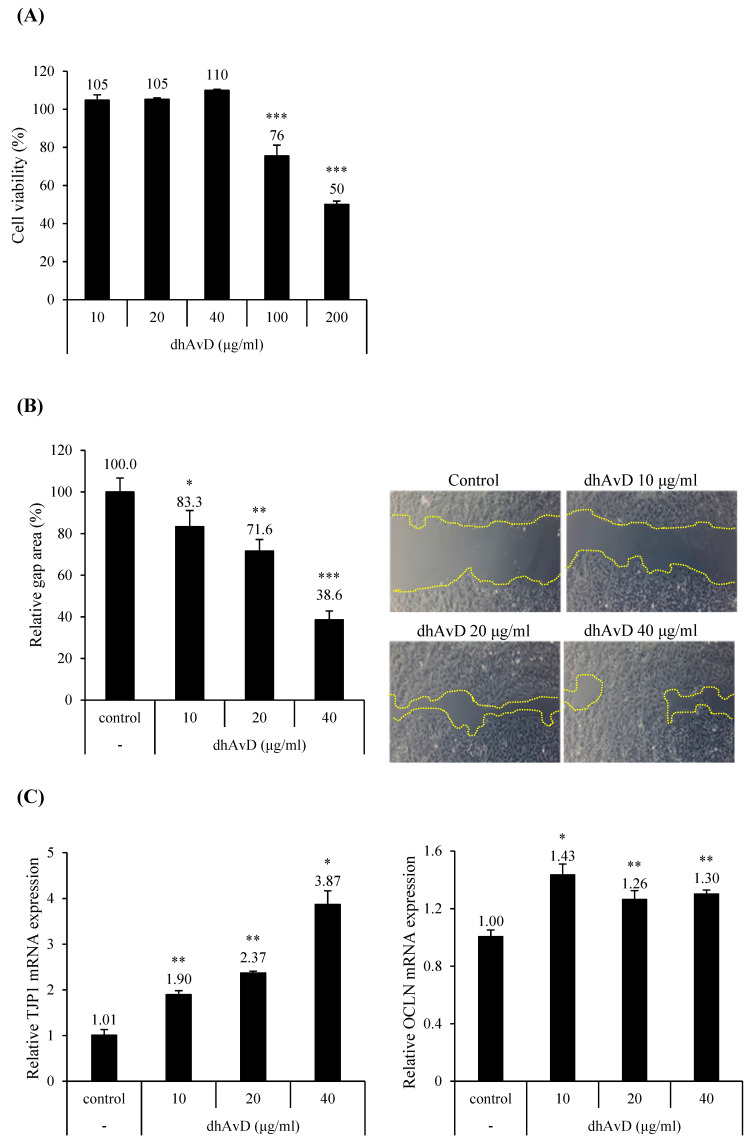

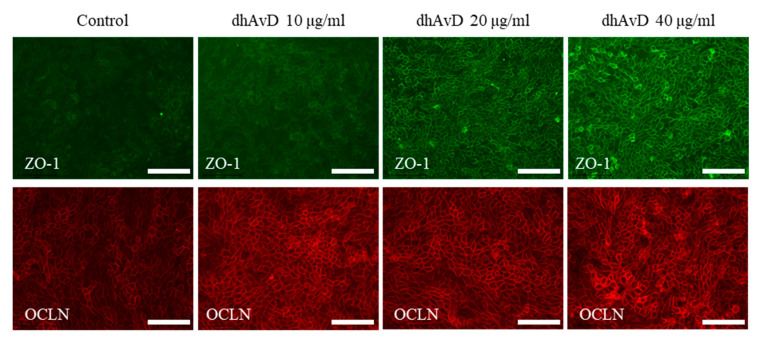
Regulation of cell proliferation and migration and mRNA and protein expression by dihydroavenanthramide D (dhAvD) on cultured human keratinocytes. (**A**) Cell viability was assessed by CCK-8 assay after 24 h treatment with dhAvD (*n* = 3). (**B**) Scratch assay was conducted and relative gap area was calculated to evaluate the effects of 24 h treatment of dhAvD (*n* = 3). (**C**) Relative mRNA and protein expressions of TJP1 and OCLN were quantified after 24 h treatment with dhAvD (scale bar = 200 µm) (*n* = 3). The expression of GAPDH mRNA expression was used as house-keeping gene. * *p* < 0.05, ** *p* < 0.01, and *** *p* < 0.001 vs. non-treated control.

To evaluate epidermal proliferative ability, we performed a CCK-8 assay by treating cultured HaCaT cells with serial concentrations of dhAvD. DhAvD treatment induced a 10% increase in proliferation up to a concentration of 40 μg/mL. However, significant cellular cytotoxicity was observed at concentrations over 40 μg/mL (Figure 3A). Further, scratch assays revealed that 40 μg/mL of dhAvD treatment for 16 h significantly accelerated wound closure by 61.4% compared to that in the control group (Figure 3B).

To evaluate the ability of dhAvD to enhance intercellular adhesion in keratinocytes, we examined the tight junction protein and mRNA expression in HaCaT cells. We observed that the treatment of dhAvD significantly increased TJP1 (ZO-1) mRNA expression up to 3.87-fold (40 μg/mL) in a dose-dependent manner, and OCLN mRNA expression up to 1.43-fold (10 μg/mL) (Figure 3C). Moreover, the protein expression of ZO-1 and OCLN was upregulated up to 2.8- and 2.2-fold, respectively (Figure 3C). Next, we examined the regulatory effect of dhAvD on several chemokines, cytokines, and cell matrix degradation factors related to skin barrier dysfunction. We also investigated the regulatory effects of dhAvD on CCL20 and IL-23, keratinocyte-derived factors that enhance Th2 cytokines that are highly expressed in atopic dermatitis [6,27,28,29], and their downstream molecules, MMP-9 and MMP-12 [30,31] (Supplementary Figure S1). Additionally, dhAvD showed synergistic effects with other chemicals (Supplementary Figure S2) [32].

### 3.3. Dihydroavenanthramide D Recovered Inflammatory Cytokine-Induced Skin Damage

**Figure 4:**
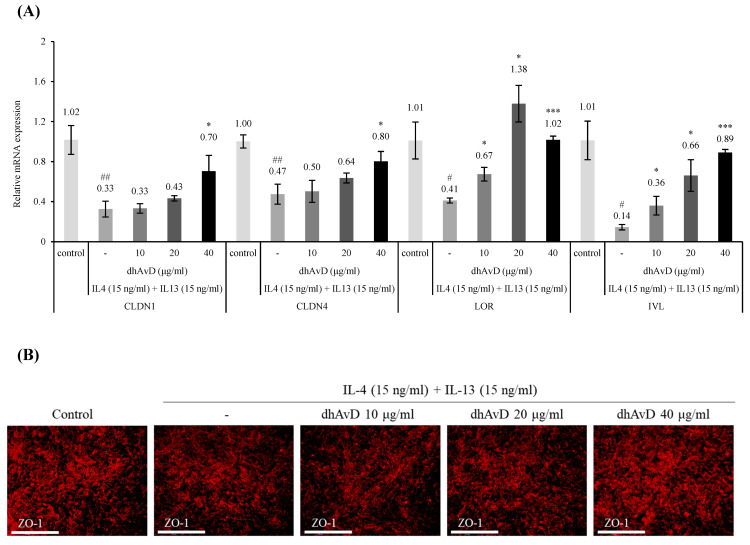
Effect of dhAvD on the mRNA and protein expressions of tight junction and differentiation markers in human keratinocytes treated with IL-4/IL-13. (**A**) mRNA levels of CLDN1, CLDN4, LOR, and IVL (*n* = 3). The expression of GAPDH mRNA expression was used as house-keeping gene. (**B**) Immunocytochemistry analysis of ZO-1 expression (scale bar = 200 μm) (*n* = 3). # *p* < 0.05 and ## *p* < 0.01 vs. non-treated control. * *p* < 0.05 and *** *p* < 0.001 vs. IL-4/IL-13-treated samples.

We investigated the barrier function recovery effect of dhAvD at the cellular level by mimicking the barrier dysfunction induced by Th2 cytokines (IL-4 and IL-13) [33,34]. Keratinocytes were co-treated with IL-4 (15 ng/mL) and IL-13 (15 ng/mL) to establish a damaged skin barrier model. IL-4 and IL-13 treatment downregulated the expression of a variety of skin barrier- and differentiation-related genes, with CLDN1, CLDN4, LOR, and IVL exhibiting the most significant reduction. To evaluate the epidermal recovery effect, HaCaT cells were co-treated with dhAvD and an IL-4/IL-13 cocktail. The mRNA expression of tight junction (CLDN1 and CLDN4) was significantly downregulated by IL-4 and IL-13 by 0.33- and 0.47-fold, respectively. The treatment with 40 μg/mL dhAvD significantly recovered CLDN1 and CLDN4 expression to 0.7- and 0.8-fold, respectively. In addition, the expression of keratinocyte differentiation markers (LOR and IVL) was significantly reduced by IL-4 and IL-13 by 0.41- and 0.14-fold, respectively. The dhAvD treatment significantly recovered LOR and IVL to 1.38-fold (20 μg/mL) and 0.89-fold (40 μg/mL), respectively. Further, dhAvD significantly recovered IL-4/IL13-induced barrier dysfunction in a dose-dependent manner (Figure 4A). Additionally, ZO-1 expression was fully recovered by co-treatment with IL-4/IL-13 and 40 μg/mL of dhAvD (Figure 4B).

### 3.4. Dihydroavenanthramide D Restored Skin Barrier Physiology and Dermal Intensity in a Whole Skin Equivalent Model

**Figure 5:**
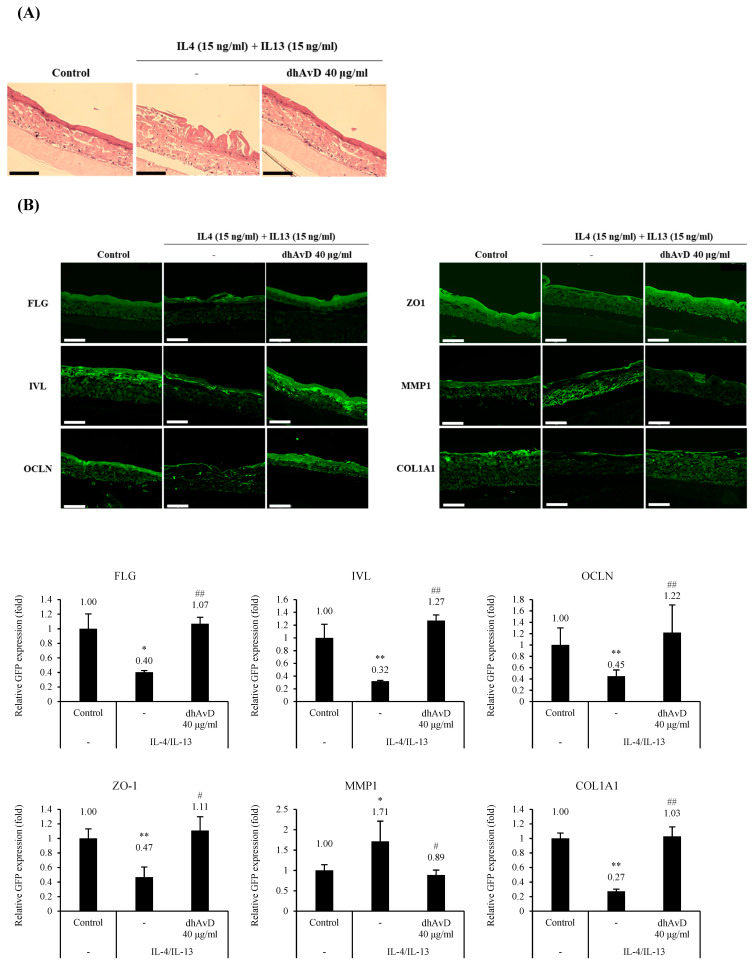
Recovery of IL-4/IL-13-induced epidermal and dermal impairment by dhAvD treatment. (**A**) Histological images of hematoxylin and eosin (H&E)-stained skin equivalents treated with IL-4/IL-13 with or without dhAvD (scale bar = 200 μm) (*n* = 3). (**B**) Immunostaining results of FLG, IVL, OCLN, ZO-1, MMP1, and COL1A1 expression in IL-4/IL-13-treated skin equivalents with or without dhAvD and their quantification (scale bar = 200 μm) (*n* = 3). * *p* < 0.05 and ** *p* < 0.01 vs. non-treated control; # *p* < 0.05 and ## *p* < 0.01 vs. IL-4/IL-13 treatment.

To further investigate the function of dhAvD in the whole skin model, we used a reconstructed human skin model (RHS) treated with IL-4 and IL-13, which are known to induce atopic dermatitis-like conditions. A histological analysis revealed that dhAvD effectively restored the thickness and density of the damaged epidermis following IL-4/IL-13 treatment. Upon the IL-4/IL-13 treatment, the epidermis exhibited a thinned and unpacked morphology resembling the skin barrier disruption observed in atopic dermatitis. Additionally, the dermis showed an overall thickening but reduced density. To further investigate these morphological changes, immunostaining was performed using several markers. (Figure 5A). Immunostaining demonstrated that dhAvD reversed the IL-4/IL-13-induced downregulation of tight junctions (OCLN and ZO-1) and epidermal differentiation markers (FLG and IVL). In contrast, dhAvD restored the skin barrier integrity by upregulating the tight junction expression, thereby strengthening intercellular adhesion and normalizing keratinocyte proliferation and differentiation, as evidenced by increased FLG and IVL expression. Moreover, in 3D skin models, dhAvD upregulated the collagen type I expression by downregulating the MMP1 expression in the dermis. This finding is consistent with the observation in Supplementary Figure S1 that dhAvD effectively inhibited MMPs secreted by keratinocytes (Figure 5B). These findings demonstrate that dhAvD effectively enhanced barrier function and improved skin elasticity in an IL-4/IL-13-induced skin barrier disruption model by strengthening intercellular adhesion, promoting epidermal cell differentiation, and inhibiting MMPs in the dermis.

### 3.5. Dihydroavenanthramide D Enhanced Skin Barrier and Reduced Skin Sensitivity In Vivo

**Figure 6:**
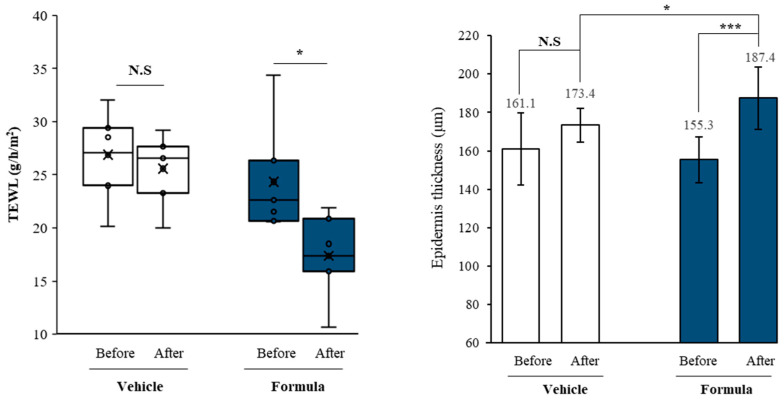
TEWL improvement and epidermal thickening effect by two-week application of a formula. * *p* < 0.05 and *** *p* < 0.001 vs. before and vehicle values (N.S: Non-Significant).

To validate the in vitro barrier-enhancing effects of dhAvD, we performed a 2-week topical application study on six individuals with sensitive skin. This study included individuals in the severe group (Table 1) and evaluated the effects of dhAvD administration on TEWL, epidermal thickness, and skin sensitivity. A formula was developed to contain a final dhAvD concentration of 24.5 μg/mL compared to the vehicle. The formula contained minimal excipients and was applied in a half-and-half test at the cheek site (Supplementary Figure S3). In contrast to the control group, which showed a non-significant decrease in TEWL from 26.8 to 25.5 after two weeks, the experimental group exhibited a significant reduction from 24.3 to 17 after two weeks. Additionally, a skin ultrasound analysis revealed that the epidermal thickness in the control group increased from 161.1 to 173.4 after two weeks, whereas the experimental group showed a significant increase from 155.3 to 187.4 after two weeks (Figure 6). Further, the glycolic acid sting test showed that overall skin sensitivity was significantly improved after a two-week application of the formula from a score of 2.33 to 0.67 (Table 3).

## 4. Discussion

This study aimed to investigate the correlation between skin barrier thickness, function, and sensitivity. Furthermore, we screened several chemicals for their ability to enhance skin barrier function and identified dhAvD as the most promising candidate (Figure 1).

Our results demonstrate a significant correlation between TEWL and epidermal thickness (Figure 2). In addition, skin sensitivity to glycolic acid was strongly correlated with TEWL and epidermal thickness (Table 1). Consistently with previous studies establishing the importance of the epidermis for healthy skin, our results suggest that a weakened skin barrier may contribute to increased skin sensitivity [35,36,37].

Sensitive skin, characterized by an impaired skin barrier and heightened susceptibility to external stimuli, is increasingly being recognized as a risk factor for premature aging. The compromised barrier and chronic inflammation associated with sensitive skin make it more susceptible to environmental aggressors and oxidative stress, thereby accelerating the aging process [38,39]. The link between sensitive skin and premature aging underscores the importance of restoring epidermal health and strengthening the skin barrier to alleviate sensitive skin symptoms and prevent the signs of aging [40].

In this study, we investigated the effects of oat-derived dhAvD on human keratinocytes. Our findings revealed that dhAvD significantly enhanced keratinocyte proliferation and migration, which are essential for barrier repair and maintenance (Figure 2A,B). Additionally, dhAvD upregulated the expression of the tight junction proteins, TJP1 and OCLN (Figure 3C), which strengthen the intercellular connections between keratinocytes, further reinforcing the skin barrier [41]. We observed a tendency for the downregulation of monocyte chemoattractant protein-1 (MCP1) and TNF-R1 expression, which are known to influence tight junction rearrangement and permeability [42], upon dhAvD treatment in our inflammatory cytokine array results. This suggests a potential mechanism for the regulation of tight junction expression mediated by inflammation. We also observed the multifaceted actions of dhAvD on the dermal matrix region. Notably, dhAvD significant potential of dermal restoration by modulating the expression of MMPs (Figure 5B, Supplementary Figure S1), consequently upregulating the expression of collagen type 1 (Figure 5B) [43].

**Figure 7:**
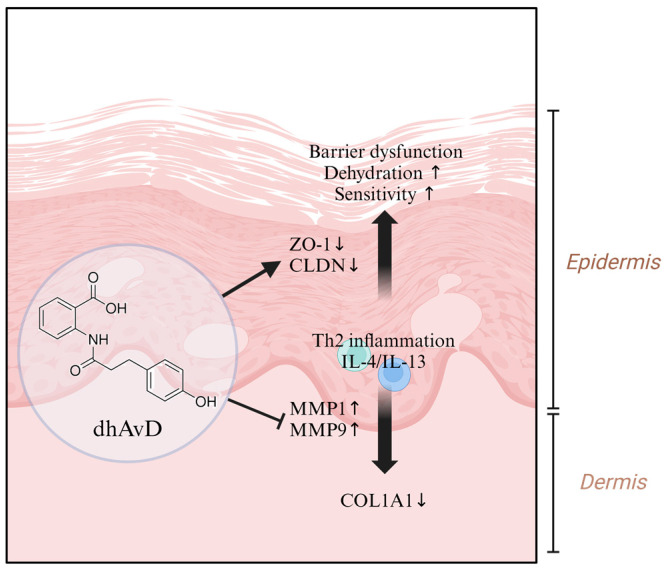
Summary of the effects of dhAvD on Th2-induced skin barrier dysfunction (↑: up-regulation, ↓: down-regulation).

The reduced skin elasticity commonly observed in various barrier-disrupted skin diseases (e.g., atopic dermatitis, psoriasis, and xerosis) is attributed not only to increased TEWL [44] but also to the influence of MMPs regulated by Th2 cytokines secreted from the epidermal basement membrane [6]. To mimic the Th2 cytokine-induced skin barrier dysfunction, we established a model by treating keratinocytes and full-thickness skin equivalents with IL-4/IL-13 [45,46]. We observed that dhAvD effectively restored the expression of essential barrier components and differentiation markers (CLDN1, CLDN4, IVL, and LOR) after treatment with IL-4/IL-13. This suggests that dhAvD may be beneficial for individuals with sensitive skin due to barrier dysfunction and inflammation (Figure 4 and Figure 5B). Also, our experimental results based on a 2-week period confirmed that dhAvD effectively recovered the skin barrier in vivo (Figure 6). Additionally, according to the literature, the topical application of formulations containing dhAvD for 8 and 12 weeks demonstrated safe anti-inflammatory and anti-itch effects on the skin as a non-steroidal facial cream, thus anticipating the long-term effects of dhAvD [47,48]. In addition, we confirmed changes in the dermis using full-thickness skin equivalents treated with IL-4/IL-13. Among several factors, the most significant change was decreased type 1 collagen due to the enhanced expression of MMP1 (Figure 5B, right below panel). Notably, dhAvD treatment not only restored barrier damage induced by IL-4/IL-13, but also decreased MMP1 expression and increased type 1 collagen expression in the dermis. Although further experiments are required, these results suggest a correlation between skin sensitivity induced by barrier dysfunction and skin elasticity. These findings indicate that barrier function restoration is crucial because prolonged damaged barrier status leads to a more rapid decline in skin elasticity compared to normal skin, called sensitive aging (Figure 7).

The ability of dhAvD to restore the damaged skin barrier induced by inflammatory stimuli is particularly noteworthy, suggesting its potential application in a wide range of skin conditions characterized by barrier dysfunction and inflammation, including pathological conditions, such as eczema, rosacea, and psoriasis, as well as in non-pathological but unhealthy skin states. Moreover, the link between a weakened skin barrier and accelerated skin aging suggests that the potential of dhAvD to improve the skin barrier function may also contribute to its anti-aging effects. Future research will reveal more specific mechanisms to fully understand the efficacy and safety of dhAvD in topical applications. While more sophisticated and detailed follow-up experiments are needed to determine whether dhAvD directly inhibits the secretion of IL4 and IL13 or suppresses tight junction degradation and collagen degradation induced by IL4 and IL13, it is clear that dhAvD plays a crucial role in inhibiting Th2-mediated barrier dysfunction.

In conclusion, this study demonstrated that the restoration of tight junction expression, suppression of inflammatory cytokines, and proliferation of the epidermis are crucial for the recovery of sensitive skin caused by skin barrier damage. Our study provides evidence that dhAvD is a promising agent that can restore damaged skin barrier and improve skin sensitivity.

## Figures and Tables

**Table 1:** Characteristics of the three groups (none, moderate, and severe).

		Number of Volunteers	Min. Age (Years)	Average TEWL	Average Epidermis Thickness
Total		39	34	22.1	167.4
Glycolic acid (GA) sting degree	None (0)	13	33	14.1	185.8
	Moderate (1)	13	32	19.7 *	169.2 *
	Severe (2–3)	13	35	32.6 ***	147.3 ***

Note: Significant differences in TEWL and epidermal thickness were observed between the severe and non-severe groups. * *p* < 0.05 and *** *p* < 0.001 versus “None” group; Student’s *t*-test.

**Table 2:** Demographic information of total subjects.

Characteristic	Description	No.	%
Gender	Male	22	56.4
	Female	17	43.6
Age	Average	34	
	Standard deviation	5.1	
	Range	27–46	

**Table 3:** Decrease in GA sting index after two-week application of dhAvD.

	Vehicle		Formula	
	Before	After	Before	After
Glycolic acid (GA) sting degree	2	1.67	2.33	0.67 **

Note: Significant differences in stinging degrees were observed before and after use. ** *p* < 0.01 versus before application; Student’s *t*-test.

## Data Availability

The datasets used and/or analyzed in this study are available from the corresponding author upon reasonable request. Some data may not have been available because of company policies and ethical restrictions.

## Supplementary Materials

The following supporting information can be downloaded at: https://www.mdpi.com/article/10.3390/cimb46090547/s1, Figure S1. Regulation of mRNA expression of inflammatory cytokine and MMPs by dihydroavenanthramide D (dhAvD) on cultured human keratinocytes. (A) Relative mRNA expression of CCL20 and IL23 was quantified after 24 h treatment of dhAvD. (B) Relative mRNA expression of MMP9 and MMP12 was quantified after 24 h treatment of dhAvD. * *p* < 0.05, ** *p* < 0.01 vs. non-treated control. Figure S2. Synergistic effect of dhAvD (24.5 μg/mL) with serine (0.05%). Based on Colby’s equation, expression of Cldn1, OCLN, ZO-1 and HAS3 protein expressions were synergitically up-regulated by dhAvD and serine. * *p* < 0.05, ** *p* < 0.01 vs. non-treated control. Figure S3. Composition of the vehicle and formula.
