# Supplementary material for: Dihydroavenanthramide D Enhances Skin Barrier Function through Upregulation of Epidermal Tight Junction Expression
Source: Curr Issues Mol Biol. 2024 Aug 23;46(9):9255–68. doi: 10.3390/cimb46090547 (PMC11430283; doi:10.3390/cimb46090547)
Supplement: Supplementary file 1 [file cimb-46-00547-s001.zip › cimb-3171209-supplementary.pdf]

Supplementary Figure S1. Regulation of mRNA expression of inflammatory cytokine and MMPs by dihydroavenanthramide D (dhAvD) on cultured human keratinocytes.
(A) Relative mRNA expression of CCL20 and IL23 was quantified after 24 h treatment of dhAvD.
(B) Relative mRNA expression of MMP9 and MMP12 was quantified after 24 h treatment of dhAvD.
* p < 0.05, ** p< 0.01vs non-treated control.

(A)

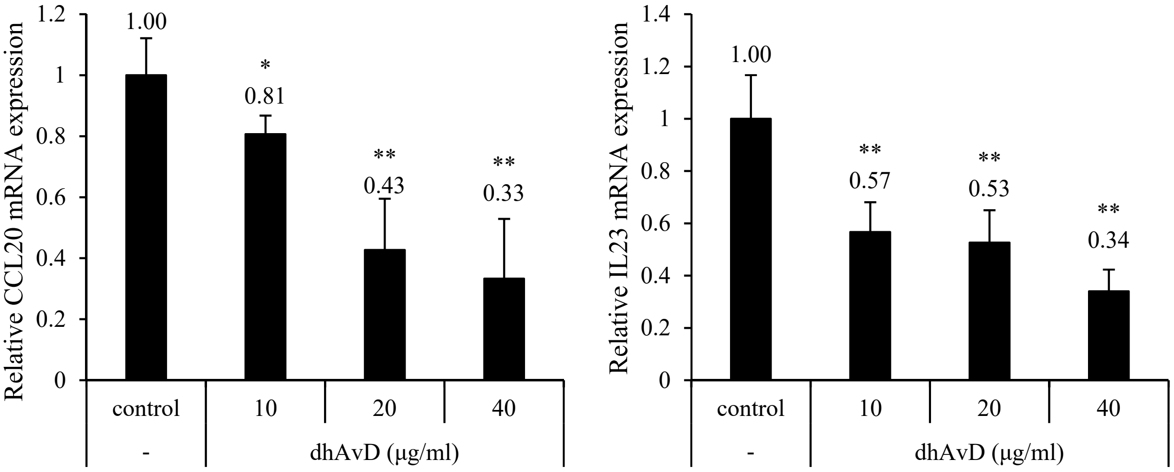

(B)

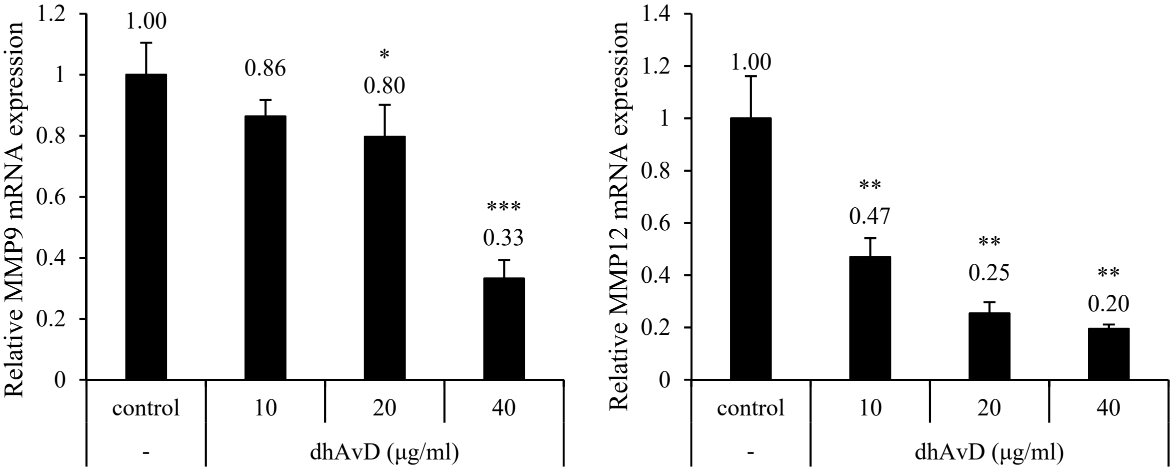

Supplementary Figure S2. Synergistic effect of dhAvD (24.5 μg/ml) with serine (0.05%). Based on Colby's equation, expression of Cldn1, OCLN, ZO-1 and HAS3 protein expressions were synergitically up-regulated by dhAvD and serine.
* $p < 0.05$, ** $p < 0.01$ vs non-treated control.

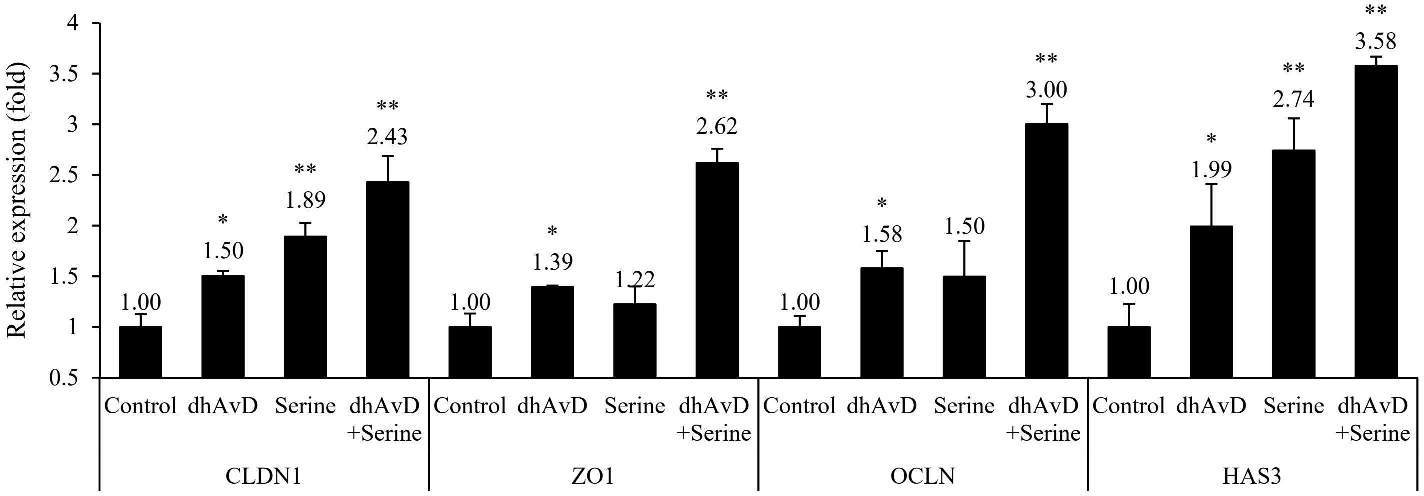

Supplementary Figure S3. Composition of the vehicle and formula.

1. Vehicle

Distilled water	90.735%
POLYQUATERNIUM	0.500%
Lauryl Dimethicone	0.300%
PROPANEDIOL	4.000%
GLYCERIN	2.000%
2,3-BUTANEDIOL	1.000%
1,2-HEXANEDIOL	1.340%
CETEARYL ALCOHOL	0.125%

2. Formula

Distilled water	90.683%
POLYQUATERNIUM	0.500%
Lauryl Dimethicone	0.300%
PROPANEDIOL	4.000%
GLYCERIN	2.000%
2,3-BUTANEDIOL	1.000%
1,2-HEXANEDIOL	1.340%
CETEARYL ALCOHOL	0.125%
Serine	0.050%
Dihydroavenanthramide D	24.5ppm
